# The role of research in evaluating and implementing digital mental health: Lecture title:

**DOI:** 10.1192/j.eurpsy.2021.60

**Published:** 2021-08-13

**Authors:** H. Riper

**Affiliations:** Clinical, Neuro And Developmental Psychology, Vrije Universiteit, Amsterdam, Netherlands

## Abstract

**Abstract Body:**

The clinical evidence and cost-effectiveness of digitalised prevention and treatment of mental disorders such as depression, anxiety and alcohol misuse have been steadily growing over the last two decades. However, bridging the gap between evidence-based eMental-health interventions and their actual delivery, evaluation and implementation in routine care has proven to be more difficult and a longer process than previously expected thereby reaching the estimated forecast of Roger’s innovation cycle of 20 years. In contrast, during the appearance of COVID-19 in 2020 for many patients and therapists digitalized treatment was the only option. Meanwhile from a scientific and policy perspective the implementation and upscaling of digital mental health care innovations in routine care have gained momentum in terms of theoretical perspectives on organizational change, empirical research into how to effectively implement digital innovations from the perspective of a variety of stakeholders and organizational levels (micro, macro and meso). In this presentation an overview of these issues will be presented, and it will be discussed whether COVID-19 might act as a turning point for the provision of large scale access to and implementation of digitalized mental health care in the near future.

**Disclosure:**

No significant relationships.

